# Monochromatic computed microtomography using laboratory and synchrotron sources and X-ray fluorescence analysis for comprehensive analysis of structural changes in bones^1^

**DOI:** 10.1107/S1600576715006214

**Published:** 2015-05-31

**Authors:** Alexey Buzmakov, Marina Chukalina, Dmitry Nikolaev, Victoriya Gulimova, Sergey Saveliev, Elena Tereschenko, Alexey Seregin, Roman Senin, Denis Zolotov, Victor Prun, Gerald Shaefer, Victor Asadchikov

**Affiliations:** a Shubnikov Institute of Crystallography RAS, Moscow, Russian Federation; b Institute of Microelectronics Technology and High Purity Materials RAS, Moscow, Russian Federation; c Institute for Information Transmission Problems (Kharkevich Institute) RAS, Moscow, Russian Federation; d Research Institute of Human Morphology, Moscow, Russian Federation; e National Research Centre ‘Kurchatov Institute’, Moscow, Russian Federation; f Department of Innovation and High Technology, Moscow Institute of Physics and Technology, Moscow, Russian Federation; g Department of Computer Science, Loughborough University, Loughborough, UK

**Keywords:** monochromatic computed microtomography, X-ray fluorescence analysis, bone

## Abstract

A combination of X-ray tomography at different wavelengths and X-ray fluorescence analysis was applied in the study of two types of bone tissue changes: prolonged presence in microgravity conditions and age-related bone growth. The proximal tail vertebrae of geckos were selected for investigation because they do not bear the supporting load in locomotion, which allows them to be considered as an independent indicator of gravitational influence. For the vertebrae of geckos no significant differences were revealed in the elemental composition of the flight samples and the synchronous control samples. In addition, the gecko bone tissue samples from the jaw apparatus, spine and shoulder girdle were measured. The dynamics of structural changes in the bone tissue growth was studied using samples of a human fetal hand. The hands of human fetuses of 11–15 weeks were studied. Autonomous zones of calcium accumulation were found not only in individual fingers but in each of the investigated phalanges. The results obtained are discussed.

## Abstract 2

Structural changes in vertebrate bone tissues caused by bone growth or by weightlessness were studied using a combination of X-ray tomography at different wavelengths and X-ray fluorescence analysis.

## 1. Introduction   

The structure of living tissues can undergo partial changes due to various diseases or in the case of extreme loads. These changes often do not have any external physiological or anatomic signs. An examination of the changes allows one to reveal the reasons for their origin. The knowledge obtained is important for diagnosis. Bone tissues are a promising indicative object for research. Results of such studies can also be used in bone-tissue engineering. Degeneration of tissues in microgravity is a serious obstacle for long-term human stays in space or for interplanetary flights. It has been found that a stay under these conditions leads to bone demineralization (Postnov *et al.*, 2003 ▶; Swinerd, 2008 ▶). Changes in the concentration of other heavier elements can also be expected. Other tissues of organisms may also have mechanosensitivity and need gravity to maintain normal metabolism. Thus, bone and muscle tissues are kinds of ‘signalling systems’, showing the earliest and most obvious response to the impact of microgravity.

Studies of vertebrates in microgravity have been conducted from the early 1990s (Besova *et al.*, 1993 ▶; Besova & Savel’ev, 1993 ▶; Grinfeld *et al.*, 1994 ▶; Savel’ev *et al.*, 1995 ▶) to the present (Roberts *et al.*, 2008 ▶; Asadchikov *et al.*, 2012 ▶; Sun *et al.*, 2013 ▶). Amphibians, reptiles and mammals have been investigated. A thorough analysis of the results allowed the following assumption. The degree of skeleton demineralization of different animals in conditions of weightlessness at the same (or approximately the same) flight duration depends on the morphological and behavioral adaptations of the investigated animals to their environment. To corroborate the assumption, both traditional histological analyses of samples and alternative techniques are used today. During the past several decades synchrotron sources have been used to determine the structure and element composition of bone tissues [see, for example, Nuzzo *et al.* (2002 ▶), Nikitin *et al.* (2007 ▶, 2008 ▶), Kharlamova *et al.* (2008 ▶), Zanette *et al.* (2012 ▶) and Deegan *et al.* (2015 ▶)]. Computed tomography affords the restoration of a spatial distribution of the linear attenuation coefficient of the probing radiation. Here, the linear attenuation coefficient is a sum of coefficients for each individual element of the composition per unit volume. For each element, the dependence of the linear attenuation coefficient on probing energy is unique and it is not linear. Hence, by changing the probing energy and by analyzing the obtained results, it is possible to estimate the elemental composition of each unit volume.

Following the reported detection of heavy element traces in the caudal vertebrae of the thick-toed gecko (Asadchikov *et al.*, 2012 ▶), we present the results of a comparative study of gecko bone tissues from different areas of its body. Gecko bone tissues from several sources, including the jaw apparatus, the spine and the bones of the shoulder girdle, are examined by computed tomography for the first time. Samples from the flight (F) group and from the delayed synchronous (S) control group were studied. First X-ray fluorescence measurements were performed. Then X-ray tomographic experiments were carried out at different energies to estimate the elemental composition distribution. The results obtained are presented. Successful comparative studies of small gecko bones showed that the technique can be applied to objects with smaller absorption. This conclusion was confirmed by several experiments. Hands of human fetuses (11–15 weeks) were studied. In each of the samples the tissues differ simultaneously in the amount of X-ray attenuation in the range from water to bone elements. Using X-ray scanning at different wavelengths allows an estimation of the distribution of major elements in the developing hand, irrespective of the amount of attenuation of each component.

The sections are organized as follows. §2 details the samples, the setups used and the reconstruction technique. The results obtained are discussed in §3 and the conclusion is given in §4.

## 2. Experimental   

### 2.1. Samples: geckos   

We used bone tissues from Turner’s thick-toed gecko (*Chondrodactylus turneri*, Gray, 1864); its most popular synonym is *Pachydactylus turneri*. We chose and investigated these reptiles as a model object for studying amniotes on the FOTON-M unmanned spacecraft numbers 2 (M2) (16 days flight in 2005 ▶) and 3 (M3) (12 days flight in 2007 ▶). Altogether 15 animals were used in each experiment: five each in the F group, the basal control group and the S group. Group M2-F spent 16 days in orbit under the following conditions: they were put in a container of 1.8 l volume, kept at an average temperature of 288–290 K, with 40–45% air moisture and at a 2 mGy radiation level. Thirty-two hours after the spacecraft landing the samples were transported to the laboratory, where they were subjected to euthanasia by injection of Nembutal into the peritoneal cavity. The basal control group was treated in the same way on the launch day. Prior to that all animals had been kept in normal conditions in a terrarium with access to water and food under a comfortable temperature of 298–306 K. Group M2-S was kept in the laboratory under the same conditions as the M2-F group and was euthanized 48 h later than the flight group. Each group consisted of one male and four female specimens. For the M3-F group most of the experimental conditions were almost the same as for M2-F. The first difference is that there were no male specimens in the groups: all five animals in each of the three groups were females. The other difference is that the average temperature maintained in the container was 293–295 K. This corresponds to an active state for the geckos rather than to winter hibernation in the first expedition. Finally, unlike the M2 expedition, the animals had access to water. After the satellite landed, the animals were transported to the laboratory 13.5 h later.

### 2.2. Samples: human fetal hand   

In a human limb the ossified bone elements are formed on the basis of replacement of the cartilage. This fact does not allow the chondral period to be considered as an appropriate period for evaluating the formation of dense bone matrix. That is why preliminary histological studies were made. The results of these studies showed that significant osteogenesis and the formation of zones of primary ossification, reaching a size suitable for the local X-ray fluorescence analysis using laboratory X-ray sources and simultaneous radiation, begins from the 11th week of fetal development. As the main interest is the analysis of the formation of primary foci of calcification of the elements of the skeleton, material from the development of human hands ranging from 11 to 15 weeks of fetal development was selected. Specialized external signs of differentiation and the most conservative brain structures were used as markers to determine the age of the fetuses (Savel’ev, 2002 ▶, 2005 ▶). Up to now the localization of primary calcification foci of the hand skeleton anlage is unknown. There is still no answer to the fundamental question of the existence of many primary calcium deposition zones or one primary area. It has been suggested that there are autonomous centers of ossification for each of the fingers, metacarpus and carpus. For this reason, the aim of our work was to identify the spatial distribution of the primary deposition foci of calcium and the associated elements in the human hand. A preliminary histological evaluation allowed us to choose skeleton samples from the right hand of human fetuses of 11–15 weeks of development. At these stages of development, it is possible to determine the main phases of primary calcium depositions in the fetal cartilage tissue, zones of cartilage resorption and secondary ossification. Material on human development had been collected from 1978 to 1985 in the territory of the USSR in accordance with the rules of the Health Ministry and stored in a special collection of the depository at the Research Institute of Human Morphology.

### 2.3. Apparatus   

**Figure 1:**
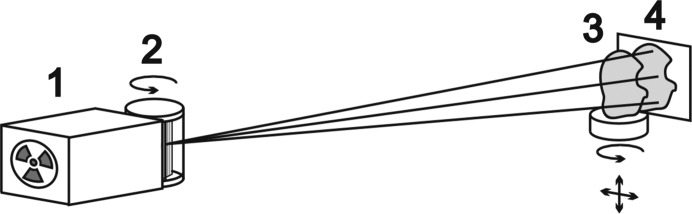
Scheme of the laboratory setup: (1) X-ray source (tube), (2) monochromator, (3) sample and (4) CCD detector.

Computed tomography systems consist of three main parts, namely a physical measuring technique, which yields integral values of a required local variable along certain paths, control and acquisition software, and a mathematical reconstruction algorithm, which calculates the local value from the collected raw data. The local value is a linear attenuation coefficient. It is a composition-dependent quantity. The results presented below were obtained in the laboratories of X-ray reflectivity and X-ray methods of analysis and synchrotron radiation at the Shubnikov Institute of Crystallography Russian Academy of Sciences (IC RAS) and on the X-ray topography and microtomography (RT-MT) beamline of the Kurchatov Synchrotron Radiation Source. To illuminate the *Z*-dependence of linear attenuation, the measurements were carried out with a parallel scanning scheme at different energies of 5.4, 8 and 10 keV. An advanced tomography system based on a diffractometer constructed at the IC RAS (Senin *et al.*, 2009 ▶) was used for 5.4 and 8 keV measurements to extract the contrast from sets of different elements. Senin *et al.* (2009 ▶) gave a detailed description of the experimental arrangement for laboratory tomography measurements. The setup is illustrated schematically in Fig. 1 ▶. An X-ray diffractometer is the basis of the setup.

A conventional X-ray tube with Mo, Cu and Cr anodes was used as a radiation source. X-ray radiation was monochromized by pyrolytic graphite to select the required wavelength. The monochromator-to-sample distance was 1 m. The sample was installed onto a goniometer. The rotation step motor was computer controlled. The exposure time was 2–10 s per projection, depending on the X-ray energy. The X-ray direct detection CCD detector with a 2 cm aperture ensures the avoidance of a layer-by-layer procedure with a resolution to 13 × 13 µm. The sample-to-detector distance was about 5 cm. The whole CT scan had an angular step of 1° over a 180° angle. To change the image contrast and to increase the linear resolution, additional measurements at 10 keV were carried out on the RT-MT beamline (Senin *et al.*, 2013 ▶). A double-crystal Ge monochromator was placed to cut the 10 keV energy line. The sample-to-CCD-detector distances were about 2.5–5 cm and the spatial resolution was 10 × 10 µm. The whole CT scan then had an angular step of 1° over a 360° angle. X-ray fluorescence analysis was carried out on an X-ray multifunctional SmartLab diffractometer with an Mo rotation anode (Shared Research Center at IC RAS). Using a focusing diffraction mirror and a Ge(220) monochromator allowed collimation and monochromatization of the probing radiation in the X-ray fluorescence measurements at 17.4 keV energy (Mo *K*α). Fluorescence spectra were registered by the energy-dispersive X-Pips detector (resolution 120 eV). To collect integral fluorescence spectra the beam used was wide enough to cover the sample entirely. The mapping of element distribution was made using a 0.25 × 0.5 mm beam.

### 2.4. Tomography reconstruction   

For image reconstruction from tomographic projections we used a regularized algebraic reconstruction technique (RegART) (Chukalina *et al.*, 2007 ▶). It searches for an answer by solving a system of algebraic equations. ART is an iterative algorithm which solves the system. Classic ART uses Kaczmarz’s method (Kaczmarz, 1937 ▶) for this. Briefly, it consists of by-turn projection of current image estimations on hyperplanes defined by each equation of the system. The procedure is repeated again if the reconstruction quality is not satisfactory. The behavior of this iterative process heavily depends on the order of the used projections (Guan & Gordon, 1996 ▶). We used the random access to projection algorithm to reduce the number of iterations at a good quality of reconstruction (van Dijke, 1992 ▶). Also the nVidia CUDA technology was used for boosting the algorithm (Buzmakov *et al.*, 2011 ▶).

## 3. Results and discussion   

### 3.1. Gecko study   

The X-ray tomography technique was used to measure gecko bone tissue samples from different locations: jaw, spine and bones of the shoulder girdle.

**Figure 2:**
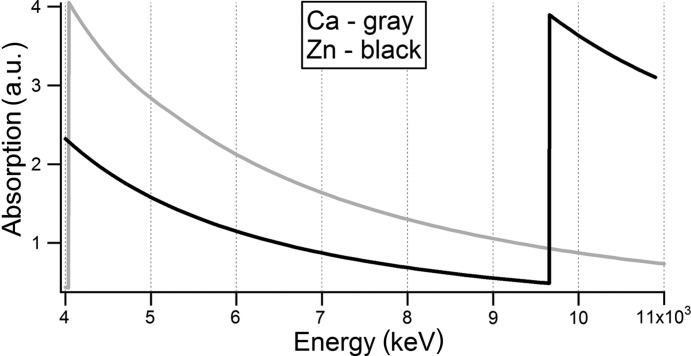
Ca and Zn absorption edges.

**Figure 3:**
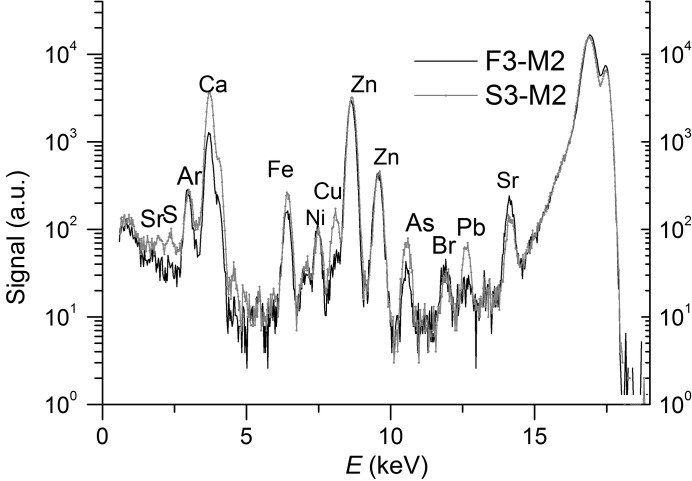
X-ray fluorescence spectra of vertebrae from geckos (M2-S3 and M2-F3).

However, the linear absorption coefficient of a reconstructed voxel is the sum of linear absorption coefficients of elements contained in the voxel. In most cases the linear combination is hard to interpret. We carried out consecutive measurements at different energies (5.4, 8 and 10 keV) to reach selectivity on the chemical composition. To illustrate the absorption contrast formation on the reconstructed images for different X-ray measurements we will turn to Table 1 ▶ and Fig. 2 ▶. Table 1 ▶ presents the values of the linear absorption coefficients for representative elements of the samples. The lists of the elements are chosen from X-ray fluorescent spectra of vertebrae samples from the geckos (Fig. 3 ▶). The number after the capital letters (S or F) corresponds to the number of the sample in the group.

Fig. 2 ▶ displays the Ca and the Zn absorption edges. The data files for graphs are taken from http://skuld.bmsc.washington.edu/scatter/AS_periodic.html. It is easy to see that at an energy of 5.4 keV calcium absorption exceeds that of zinc by one and a half times. At 10 keV the picture is completely opposite. The Zn absorption is four times larger than the absorption by Ca. That is, if our sample contained only calcium and zinc, we could estimate unambiguously the contents of each, analyzing the results at 5 and 10 keV.

**Figure 4:**
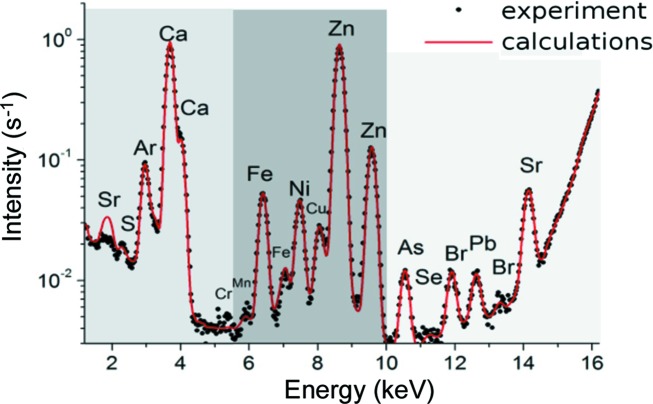
Fluorescence spectrum: divided into three zones according to the deposits in the process of absorption.

**Figure 5:**
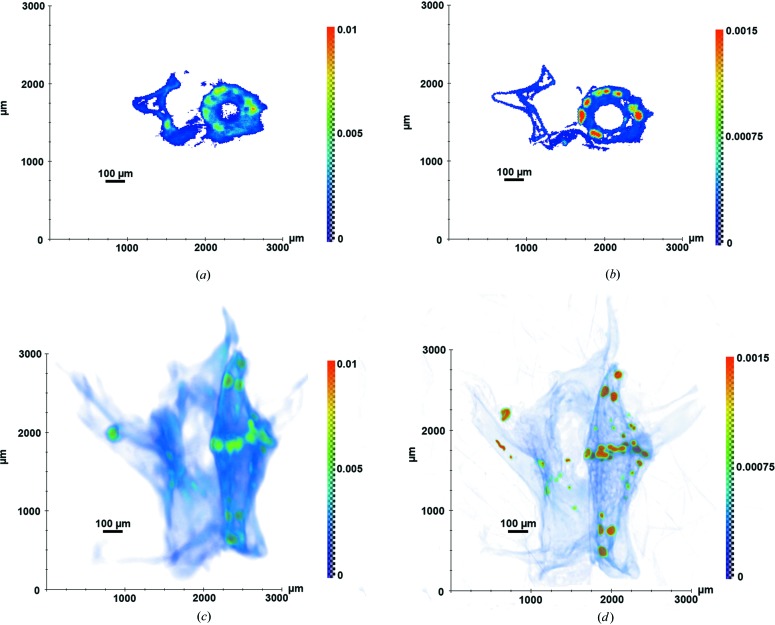
X-ray tomography reconstruction of gecko vertebra M2-S4 at 5.4 keV (*a*), (*c*) and at 10 keV (*b*), (*d*): magnified cross sections (*a*), (*b*) and volume images (*c*), (*d*).

**Figure 6:**
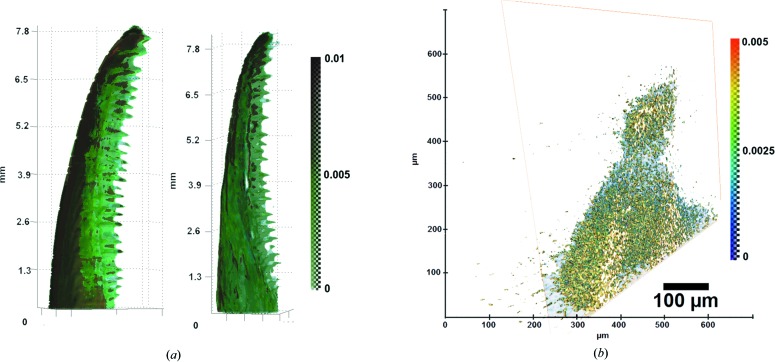
(*a*) X-ray tomography reconstruction of gecko jaws at 8 keV and (*b*) the result of a reconstruction of a gecko tooth using Bragg magnifiers.

But, the samples analyzed have a more complicated composition. Therefore, we united elements in groups, according to Fig. 4 ▶, to analyze their space distribution in the reconstructed images like those in Fig. 5 ▶. If we consider the spectrum (Fig. 4 ▶) from left to right, the first group of elements provides the greatest contribution to the tomographic projection contrast at 5.4 keV energy (Figs. 5 ▶ *a* and 5*c*), the second one at 8 keV energy (Fig. 6 ▶ *a*) and the third one at 10 keV energy (Figs. 5*b* and 5*d*). Two spectra (M2-S3 and M2-F3) are presented in Fig. 3 ▶.

The beam was wide enough to cover a sample entirely. Samples were placed on polished silicon and sapphire plates with a known chemical composition. These experimental conditions (angle of incidence is 0.25° and 0.1 × 10 mm slits are placed in front of the sample) allowed the minimization of the contribution from the substrates to a registered signal. The *PyMCA* program with GNU General Public License version 2.0 (GPLv2) was used for X-ray fluorescence data analysis (Figs. 3 ▶ and 4 ▶). Experiments were carried out in air, which gave rise to the peak of argon. The presence of an Ni component is due to the design of the detector.

A comparison of the spectra for the F and the S series for both experiments on FOTON-M (M2 and M3) does not reveal strong bone-tissue demineralization. This could be due to the fact that the geckos were attached to the container surfaces during most of the flight. Minor differences are caused by two reasons. The fluorescence source distributions inside the vertebrae are not identical owing to the presence of individual features of the objects. The non-identical shape of the vertebrae causes different lengths for the optical paths of fluorescent radiation and hence a difference in the attenuation degree.

Fig. 5 ▶ presents the X-ray tomography reconstruction of the vertebra of a gecko (sample M2-S4) at two different energies. Unlike the Table 1 ▶, the voxel values in the figures are given in mm^−1^. Several homogeneous regions of interest (high-absorbing and low-absorbing regions) on the reconstructed images were analyzed to calculate the relative errors (Dawood *et al.*, 2012 ▶) for each energy. The results show that the errors do not exceed 10% in the region of interest reviewed. Because the differences in the absorption contrast in full volume images are hard to analyze, magnified cross sections are also presented in Fig. 5 ▶. Comparison of the two cross sections showed that fluctuations in the distribution of the main components in bone tissues are clearly observed at 5.4 keV. At high energy this is enough information to describe the position and the size parameters of the inclusions. However, to describe the precise composition of each individual inclusion, the information is still not sufficient.

In Fig. 6 ▶(*a*) a reconstruction of the jaws of two geckos is presented. The measurements were made at 8 keV.

To increase the spatial resolution we used a pair of crossed asymmetric cut single crystals (Bragg magnifiers). Each crystal magnifies only in one direction; therefore, a pair is used. Crystals were cut with the asymmetry coefficient 15 for 8 keV. Both crystals were set to the maximum of the rocking curve, as any detuning from the peak position primarily influences the spatial resolution. The scheme of this setup is similar to that in Fig. 1 ▶, but asymmetric cut crystals were used instead of the monochromator. When calculating the value of the attenuation coefficient, we used the normalization on the X-ray beam measured after passing through the two crystals in the absence of the studied sample according to the Beer–Lambert–Bouguer law. The tooth of the same gecko jaw was chosen as a test object. In Fig. 6 ▶(*b*) a reconstructed image of its structure with ∼1 µm voxel size is displayed. The image is computer sectioned to show its inner structure.

Analysis of the measurement results of bone tissues from different parts of the gecko body did not reveal any great differences in the composition of the basic components of the bone tissue from different sources. Note that the artifacts in the reconstructed images have two sources. The first are artifacts of the reconstruction method. The second are related to the structure of the polypropylene holder which is attached to the samples.

### 3.2. Human fetal hand study   

**Figure 7:**
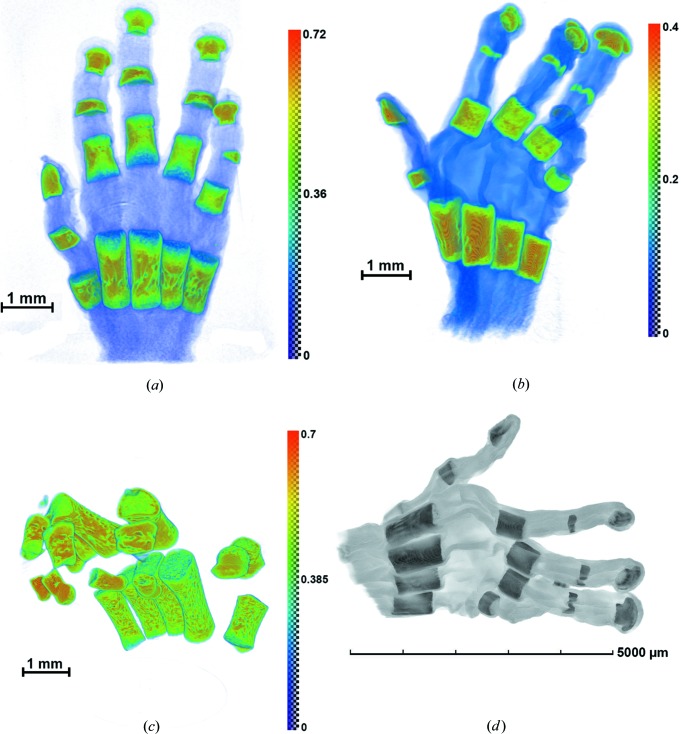
(*a*)–(*c*) Results of a reconstruction of human fetus hands (11–15 weeks). The wavelength of X-ray radiation is λ = 0.5 Å. (*d*) Reconstruction obtained on the X-ray microtomograph Xradia VersaXRM-500.

**Figure 8:**
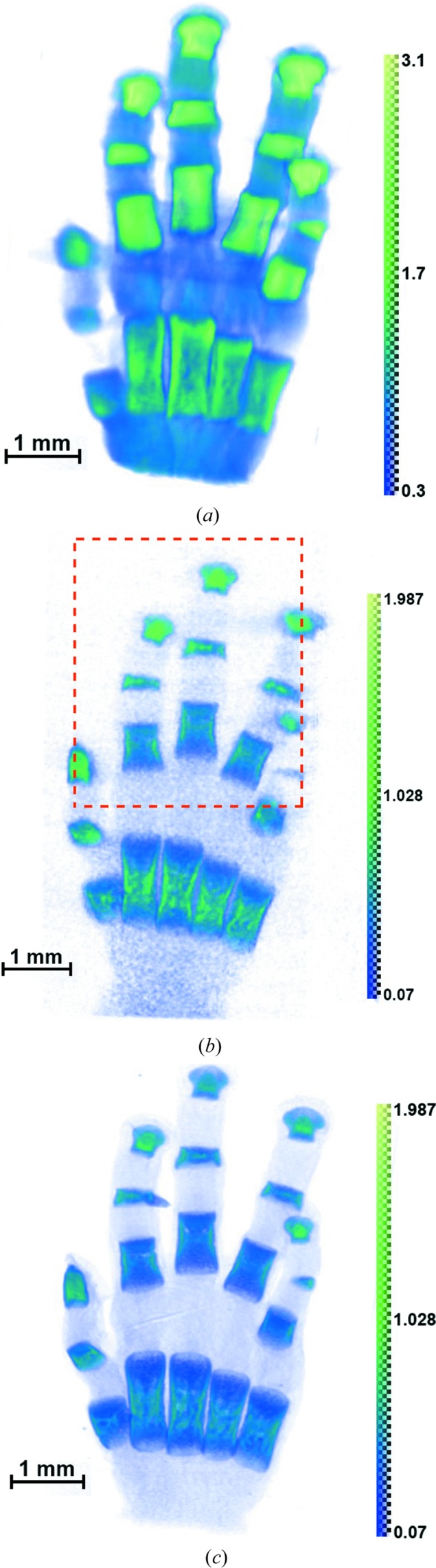
Reconstruction projections of a human fetus hand: 13 week sample using different wavelengths of X-ray radiation. (*a*) λ = 1.54 Å, (*b*) λ = 0.71 Å, (*c*) λ = 0.5 Å. The dotted line square encloses the fluorescent mapping region of interest discussed in the text.

**Figure 9:**
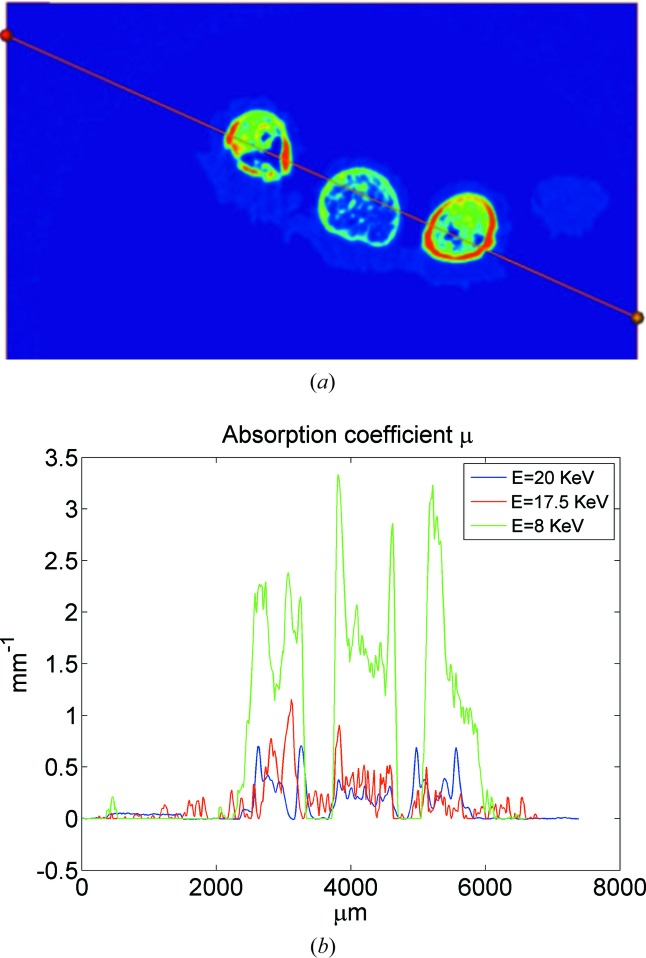
(*a*) One of the tomographic sections of a sample of fetal hand bone and (*b*) the absorption ratio at different wavelengths.

Samples of bones from human fetus hands were studied on the laboratory X-ray microtomography setup and on a multifunctional diffractometer with a rotating anode and fluorescence setup at the Shubnikov Institute of Crystallography RAS, and on the synchrotron station ‘RT-MT’ at the Kurchatov Synchrotron Center at the energies 8, 17.5 and 20 keV (corresponding to the wavelengths 1.54, 0.71 and 0.5 Å). One of the samples was also studied using an X-ray microtomograph Xradia VersaXRM-500 (http://www.xradia.com) at an accelerating voltage of 60 keV and a spatial resolution of ∼2 µm. In this case, a polychromatic spectrum of radiation does not allow the acquisition of a true value of the linear absorption coefficient μ, although it does provide object reconstruction details of high quality. Typical results of the reconstruction are shown in Fig. 7 ▶. Fig. 8 ▶ presents the results of a reconstruction of one of the objects (13 week sample) at different wavelengths. This figure illustrates the significant changes in the absorbing contrast caused by changing the wavelength of the probing radiation. In Fig. 9 ▶ the section of one of the samples and the ratio of its absorption at different wavelengths are shown. We see (Fig. 9 ▶ *b*) that a change in wavelength in different parts of bone absorption varies non proportionally. This fact, probably, indicates that high absorption elements (of higher atomic numbers) are primarily concentrated in dense areas of the bone tissue.

**Figure 10:**
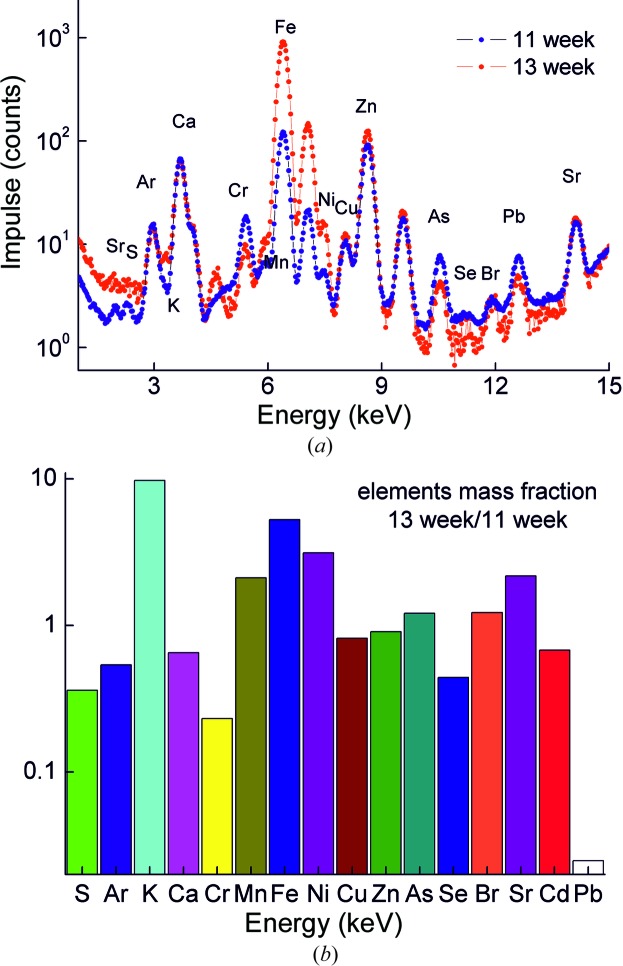
(*a*) Fluorescence spectra from the 11 and 13 week samples. (*b*) Comparison of mass fraction of the elements in the 11 and 13 week samples.

**Figure 11:**
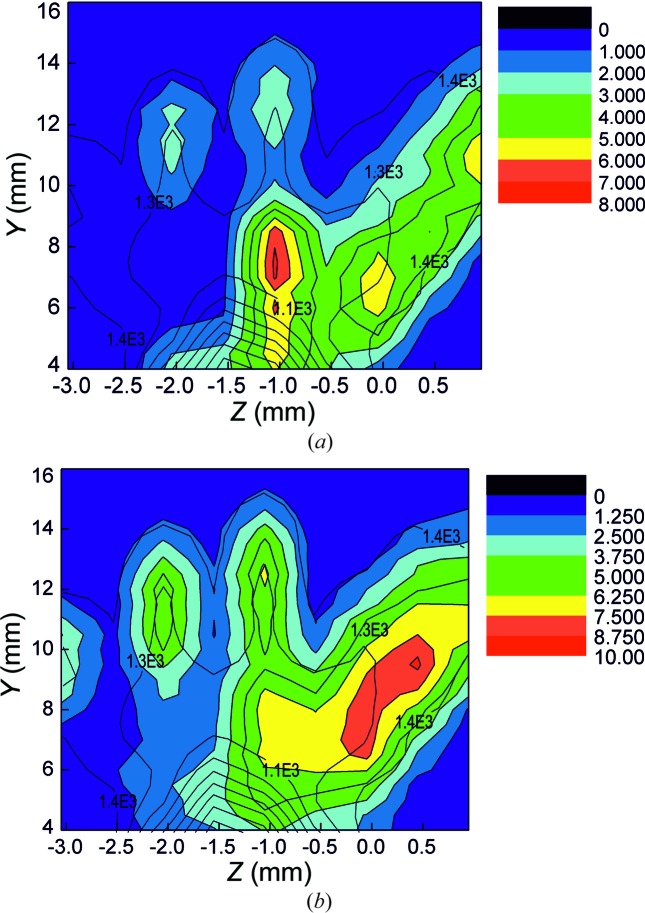
Mapping of (*a*) Ca and (*b*) Zn distributions in the 13 week sample.

The composition of the samples includes elements in a wide range (Fig. 10 ▶ *a*). Note that significant differences in the mass fraction of some elements can be explained by individual variability of the samples. The element distribution inside the samples was studied by X-ray fluorescence mapping. A comparison of the Ca (as the matrix element) and the Zn distribution [Figs. 11 ▶(*a*) and 11(*b*), correspondingly] shows that their localizations are different. For comparison with the tomography data, the fluorescence mapping region is shown on Fig. 8 ▶ enclosed by the dotted line square. The measurement time is 5 min at each point. Fluorescence yield intensity is presented in counts per second.

We can state that the greater part of the heavy element content is concentrated inside the densest part of the samples.

## 4. Conclusion   

In this paper, we demonstrate the applicability of the combination of X-ray tomography at different wavelengths and X-ray fluorescence analysis for the identification of differences in bone tissue. It was shown for the first time that gecko bone tissues from a variety of sources, including the jaw apparatus, spine and bones of the shoulder girdle, have no significant differences in their composition in spite of strong differences in the structure. Studies have shown that the peripheral parts of the samples contained no elements with atomic numbers greater than Ca or their concentration is smaller than the sensitivity of the tomographic technique. This is consistent with our earlier data obtained by scanning electron microscopy. For the vertebrae of geckos no significant differences were revealed in the elemental composition of flight (F) samples and synchronous (S) samples within each FOTON-M experiment (M2 and M3). Hands of human fetuses (11–15 weeks) were also studied. The differentiation of both bones of the hand and the phalanges of individual fingers occurs between the 11th and 15th weeks. At this period, the alternation of loose sections consisting of mesenchymal cells with anlages of cartilage and later the bone occurs. Our analysis of the dynamics of human hand development from 11 to 15 weeks of ontogenesis showed that outside the zones of extracellular matrix (Ca, P) formation a previously unknown morphological process occurs which predicts the direction of osteoblast differentiation. Autonomous zones of calcium accumulation were found not only in individual fingers but in each of the investigated phalanges. These data were confirmed by local X-ray fluorescence analysis.

## Figures and Tables

**Table 1:** Linear absorption coefficients Data taken from http://www.csrri.iit.edu/periodic-table.html in December 2013.

Element	Atomic number (*Z*)	Absorption coefficient (cm^1^) (at 5.4keV)	Absorption coefficient (cm^1^) (at 8keV)	Absorption coefficient (cm^1^) (at 10keV)
Ca	20	780	270	145
Fe	26	896	2376	1325
Cu	29	1397	467	1961
Zn	30	1233	430	1738
As	33	1292	439	237
Sr	38	868	297	160
